# Regulation of Bax-dependent apoptosis by mitochondrial deubiquitinase USP30

**DOI:** 10.1038/s41420-021-00599-6

**Published:** 2021-08-11

**Authors:** Ding Yan, Xiaofen Li, Qianqian Yang, Qingtian Huang, Leyi Yao, Peiquan Zhang, Wenshuang Sun, Shuhui Lin, Q. Ping Dou, Jinbao Liu, Xin Chen

**Affiliations:** 1 Affiliated Cancer Hospital & Institute of Guangzhou Medical University, Guangzhou, 510095 China; 2 Guangzhou Municipal and Guangdong Provincial Key Laboratory of Protein Modification and Degradation, School of Basic Medical Sciences, Guangzhou Medical University, Guangzhou, 511436 China; 3 Barbara Ann Karmanos Cancer Institute and Departments of Oncology, Pharmacology & Pathology, School of Medicine, Wayne State University, Detroit, MI 48201 USA

**Keywords:** Cancer, Cancer

## Abstract

Deubiquitinates (DUBs) have been suggested as novel promising targets for cancer therapies. Accumulating experimental evidence suggests that some metal compounds have the potential to induce cancer cell death via inhibition of DUBs. We previously reported that auranofin, a gold(I)-containing agent used for the treatment of rheumatoid arthritis in clinics, can induce cell death by inhibiting proteasomal DUBs in a series of cancer cell lines. Unfortunately, currently available gold compounds are not potent in inhibiting DUBs. Here, we report that: (i) aumdubin, a synthetic derivative of auranofin, exhibited stronger DUB-inhibiting and apoptosis-inducing activities than auranofin in lung cancer cells; (ii) aumdubin shows high affinity for mitochondrial DUB USP30; (iii) aumdubin induces apoptosis by increasing the ubiquitination and mitochondrial location of Bax protein; and (iv) USP30 inhibition may contribute to Bax-dependent apoptosis induced by aumdubin in lung cancer cells. These results suggest that gold(I)-containing agent aumdubin induces Bax-dependent apoptosis partly through inhibiting the mitochondrial DUB USP30, which could open new avenues for lung cancer therapy.

## Introduction

Global epidemiological data identified lung cancer as the second frequently diagnosed cancer and the most frequent cause of death from cancer in men [1]. Regardless of recent dramatic decline in its mortality, lung cancer still caused more deaths than the combination of breast, prostate, colorectal, and brain cancers [2]. Conventional chemotherapy is one of the currently used methods of treating lung cancer, however, its effect is unsatisfactory as various side effects and drug resistance are observed clinically. Therefore, alternatives such as targeted therapies are needed for improving the current lung cancer treatment.

Apoptosis, a very tightly programmed cell death plays a critical role in normal cell development and human diseases, such as cancer. Evading apoptosis is one of the hallmarks of cancer that leads to excessive proliferation and acquired resistance to therapeutic agents [3]. Therefore development of therapeutic compounds to induce apoptosis in tumor cells is highly significant and urgently needed [4]. Mitochondria act as the core regulator of the initiation of apoptosis triggered by intrinsic death signals. Upon apoptotic stimuli, Bax proteins are activated, then oligomerize and form pores on mitochondrial outer membrane. The permeabilization of the mitochondrial outer membrane by Bax is essential for initiating mitochondrial apoptosis through triggering the release of pro-apoptosis factors from mitochondria. Post-translational modifications (e.g., phosphorylation and ubiquitination) play crucial roles in the regulation of the function and stabilization of Bax proteins. Phosphorylation of Bax at serine184 mediated by kinases (e.g., AKT and PKCzeta) regulates the translocation of Bax to mitochondria [5–7]. Some E3 ligases (e.g., parkin and IBRDC2) can regulate the ubiquitination and function of Bax [8, 9]. However, the enzymes required for deubiquitination of Bax have not been conclusively identified.

Deubiquitinates (DUBs) comprise a family of proteases that regulate the process of deubiquitination, and are considered as a novel target for developing anticancer therapeutics [10]. For example, genetic and functional studies have shown that two proteasomal DUBs, USP14 and UCHL5, play a regulatory role in the control of tumor cell proliferation and survival [11–14]. USP30, a DUB present in the mitochondrial outer membrane, plays an important role in the regulation of mitochondrial morphology and mitophagy [15, 16]. USP30 promotes stabilization of dynamin-related protein 1 (DRP1) or ATP citrate lyase (ACLY), resulting in accelerated development of hepatocellular carcinoma [17, 18]. USP30 depletion promotes ubiquitylation of TOM20 and depolarization‐induced cell death [19]. In addition, USP30 can be located to peroxisomes, where it regulates the process of pexophagy [20, 21]. Although many studies have been focused on the function of UPS30 in deubiquitination of mitochondrial proteins, its role in mitochondrial apoptosis remains largely unclear.

Metal complexes have been gaining increasing attention recently due to their strong DUB-inhibiting effects [22]. We previously reported that a gold-containing compound, auranofin, inhibited proteasome-associated DUBs with promising anti-tumor effects in several cancer cell lines [23, 24]. In the current study, we provide evidence that aumdubin, a new auranofin derivative, inhibits the mitochondrial outer membrane DUB USP30 and consequently induces Bax-dependent mitochondrial apoptosis in human lung cancer cells in vitro and in vivo.

## Results

### Aumdubin is a potent inhibitor of several DUBs including USP30

**Fig. 1:**
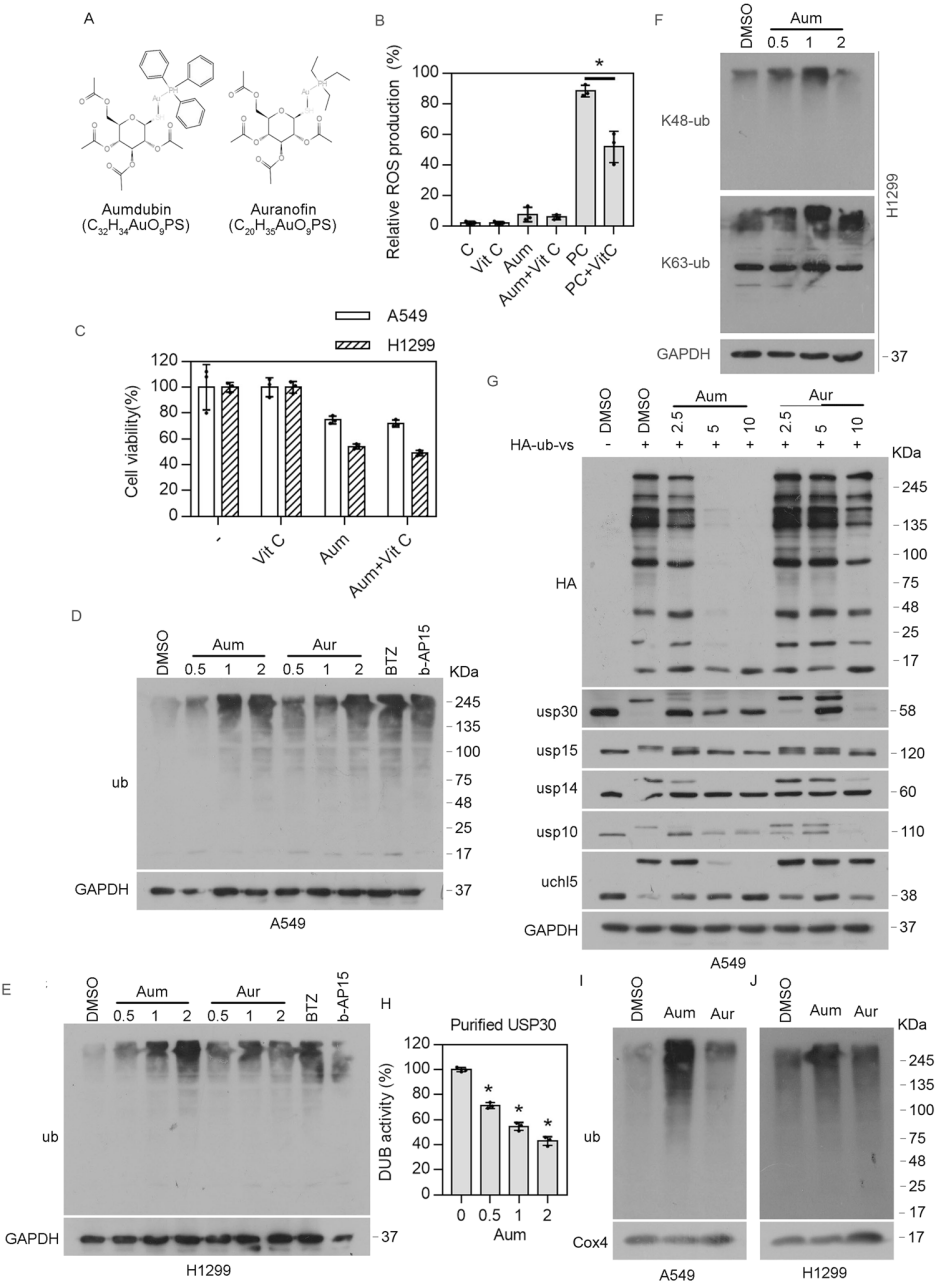
Aumdubin inhibits USP30 stronger than auranofin in A549 and H1299 cells. **A** Chemical structure of aumdubin and auranofin. **B** Aumdubin did not induce the production of ROS. H1299 cells were exposed to aumdubin (1 µM) or positive control ROSup (PC) in the absence or presence of antioxidant vitamin C (Vit C, 100 µM) for 1 h, ROS was detected with DCF-DA staining by flow cytometry. C: control. Mean ± s.d. (n = 3). ^*^P < 0.05. **C** Cytotoxic effects of aumdubin may be not dependent on ROS production. A549 and H1299 cells were exposed to aumdubin (1 µM) in the absence or presence of antioxidant vitamin C (Vit C, 100 µM) for 24 h, and then were subjected to MTS assay. Mean ± s.d. (*n* = 3). **D**, **E** Accumulation of ubiquitinated proteins by aumdubin. A549 (**D**) and H1299 (**E**) cells were treated with various concentrations of aumdubin (Aum) and auranofin (Aur) for 6 h, and then total ubiquitinated proteins were detected with western blotting assay. Bortezomib (BTZ, 100 nM) and b-AP15 (0.5 μM) were used as positive controls. GAPDH were used as a loading control. **F** Accumulation of K48- and K63-linked ubiquitinated proteins by aumdubin. H1299 cells were treated with various concentrations of aumdubin (Aum) for 6 h, and then K48- and K63-linked ubiquitination were detected with western blotting assay. GAPDH were used as a loading control. **G** Aumdubin inhibits DUBs. A549 cells were treated with the indicated concentrations of aumdubin and auranofin for 3 h, and then whole-cell lysates were labeled with HA-ub-vs and immunoblots with the indicated antibodies. **H** Aumdubin inhibits USP30. The purified recombinant USP30 was treated with the indicated concentration of aumdubin for 6 h, followed by detecting DUB activity with Ub-AMC reagent. **I**, **J** Aumdubin induces ubiquitination of mitochondrial proteins. A549 (**I**) and H1299 (**J**) cells were treated with the indicated concentrations of aumdubin and auranofin for 6 h, and then mitochondrial proteins were isolated using mitochondrial protein extraction kit. Ubiquitinated proteins were detected with western blotting assay. COX4 were used as a loading control.

Gold(I)-containing agent auranofin has been shown to induce reactive oxygen species (ROS) production and subsequent cell death through inhibition of thioredoxin reductases (TrxR) [25]. In addition, our previous studies have shown that treatment with auranofin induced proteasomal DUB inhibition in a series of cancer cell lines [24]. A strategy for mitochondria-targeting small molecules is the modification of lipophilic cationic moieties such as triphenylphosphonium (TPP), which allows the lipophilic cationic-linked compounds to specifically permeate phospholipid bilayers into the “negative” mitochondria matrix [26]. To develop more specific mitochondrial DUB inhibitors, we synthesized a derivative of auranofin, named as aumdubin, by the replacement of TPP group with ethyl group, as shown in Fig. 1A. We first tested whether aumdubin could induce ROS by targeting TrxR in lung cancer cells. Interestingly, aumdubin has minimal effects to induce the increase of ROS in HT1299 cells (Fig. 1B). Consistently, aumdubin-induced cytotoxicity was not inhibited by a ROS scavenger vitamin C (Fig. 1C), which is an effective inhibitor of ROS in H1299 cells (Fig. 1B). Thus, TrxR may not be the main target of aumdubin in lung cancer cells. Next, we hypothesized that aumdubin might be able to inhibit the mitochondrial DUBs. To test this hypothesis, we determined the effects of deubiquitination inhibition abilities of aumdubin, and found that aumdubin, similar to auranofin, markedly increased the ubiquitination of intracellular proteins in A549 and H1299 cells (Fig. 1D, E), indicating that aumdubin is able to inhibit the activity of intracellular DUBs. We also found that aumdubin treatment increased the levels of both K48- and K63-linked ubiquitination (Fig. 1F). In addition, DUB binding assay was performed by HA-Ub-vs probe labeling, as previously described [27]. We found that aumdubin exhibited stronger binding with several tested DUBs (including USP30, USP14, USP10, and UCHL5) than auranofin, as shown in Fig. 1G. In particular, aumdubin (2.5 μM) was able to completely inhibit the binding of mitochondrial USP30 with HA-Ub-vs (Fig. 1G). In contrast, aumdubin have weak binding with USP15 (Fig. 1G), a DUB also located in mitochondria and having functions similar to USP30 in the deubiquitylation of mitochondrial proteins [28]. Consistently, aumdubin inhibits the DUB activity of purified USP30 in a dose-dependent manner (Fig. 1H). Since USP30 is a mitochondrial DUB, we then examined the effects of aumdubin on the ubiquitination level of mitochondrial proteins. As shown in Fig. 1I, J, aumdubin, but bot auranofin, induced the accumulation of ubiquitinated mitochondrial proteins in A549 and H1299 cells. Taken together, these results indicate that aumdubin inhibits DUBs, including mitochondrial USP30, in lung cancer cells.

### Aumdubin exhibits greater anti-tumor activity than auranofin

**Fig. 2:**
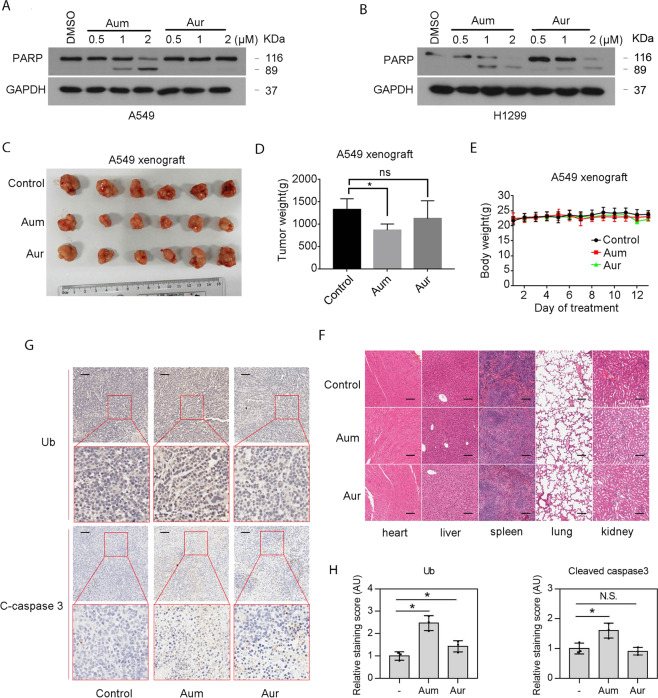
Aumdubin exhibits stronger anti-tumor activities than auranofin in vitro and in vivo. **A**, **B** Aumdubin-induced apoptosis is stronger than auranofin. A549 (**A**) and H1299 (**B**) cells were treated with various concentrations of aumdubin (Aum) or auranofin (Aur) for 24 h. PARP were analyzed with western blotting. GAPDH were used as a loading control. Nude mice bearing A549 xenograft tumors were intraperitoneally treated with vehicle (DMSO:Kolliphor:normal saline = 1:3:6), or aumdubin (Aum) or auranofin (Aur) (8.8 μmol/kg) everyday for 13 days after inoculation of A549 cells. Tumor image (**C**), tumor weight (**D**), body weight (**E**), and histopathology (**F**) were shown. Mean ± s.d. (*n* = 6). ^*^P < 0.05. Representative micrographs (**G**) and relative quantification (**H**) of immunohistochemical staining of tumor tissue for ubiquitinated proteins (Ub) and cleaved caspase 3 in nude tumor tissues was shown. Scale bars, 50 µm. Mean ± s.d. (*n* = 3). ^*^P < 0.05.

We also found that compared to auranofin, aumdubin shows greater activity to induce apoptosis in vitro, as indicated by poly-ADP-ribose polymerase (PARP) cleavage (Fig. 2A, B) in cultured lung cancer cells. We next tested the effects of aumdubin on the growth of lung tumor cells in vivo. Nude mice xenograft models of A549 cancer cells were established and treated with the same molar dose of aumdubin or auranofin. We found that aumdubin exhibited stronger anti-tumor activities than auranofin. Compared with the control group, aumdubin treatment significantly reduced the tumor size and tumor weight, whereas auranofin group had only moderate effect (Fig. 2C, D). Importantly, there was no obvious difference in the body weight of the nude mice and changes in hematoxylin-eosin staining of organs (such as heart, liver, spleen, lung, and kidney) among these three groups (Fig. 2E, F), indicating that aumdubin at the used dose has no toxicity. Moreover, DUB inhibition and apoptosis induction in xenograft tissues were also observed by immunohistochemistry staining of ubiquitinated proteins and cleaved caspase 3 in the aumdubin group compared with controls (Fig. 2G, H). Collectively, aumdubin exhibited stronger anti-tumor activities than auranofin in vitro and in vivo.

### Aumdubin induces mitochondrial apoptosis

**Fig. 3:**
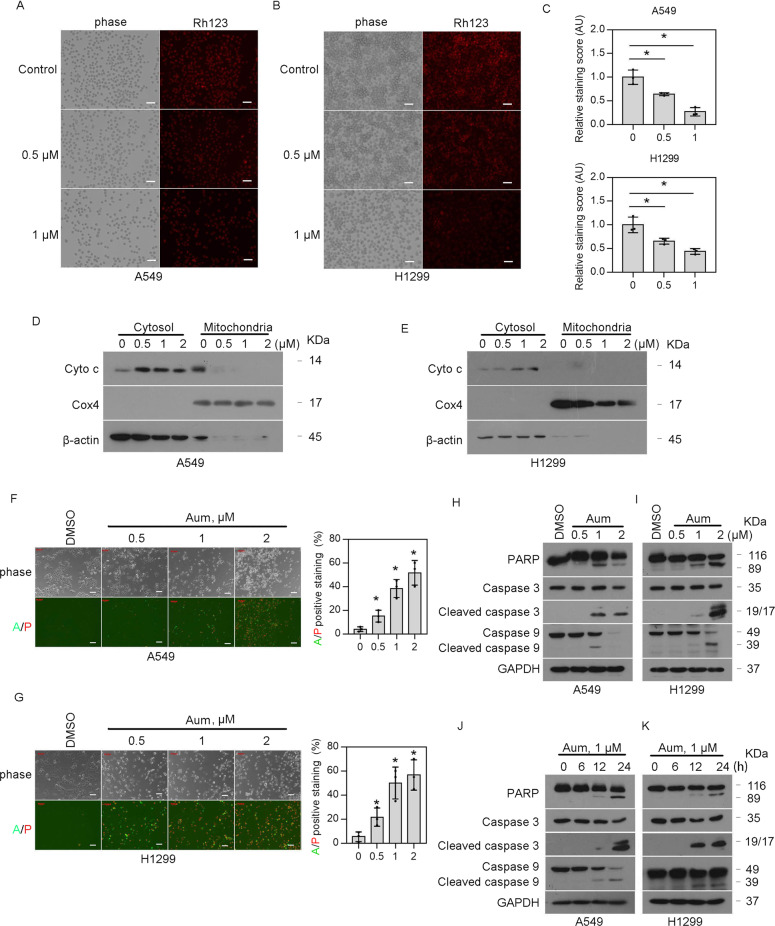
Aumdubin induces mitochondrial apoptosis in A549 and H1299 cells. **A**–**C** Aumdubin induces downregulation of mitochondria membrane potential. A549 and H1299 cells were treated with aumdubin for 12 h, and then mitochondrial membrane potential were detected by rhodamine-123 (Rh123) staining, following imaged with a fluorescence microscope. The representative images (**A**, **B**) and relative quantification (**C**) were shown. Scale bars, 50 µm. Mean ± s.d. (*n* = 3). ^*^P < 0.05. **D**, **E** Aumdubin induces the release of cytochrome c from mitochondria into cytoplasm. A549 (**D**) and H1299 (**E**) cells were treated with various concentrations of aumdubin for 24 h. Cytochrome c (Cyto c) was analyzed with western blotting. COX4 and β-actin were used as loading control for mitochondria and cytosol, respectively. **F**, **G** Aumdubin induces apoptosis. A549 (**F**) and H1299 (**G**) were treated with different doses of aumdubin (Aum) for 24 h, then apoptotic cells were detected by Annexin V-FITC/propidium iodide (PI) double staining, and the stained cells were recorded using an inverted fluorescence microscope. Scale bars, 50 µm. The relative quantification of apoptotic cells were shown. **H**–**K** Aumdubin induces the activation of caspases. A549 and H1299 cells were treated with either various concentrations of aumdubin for 24 h (**H**, **I**) or the indicated concentrations of aumdubin for various time (**J**, **K**). PARP and caspase-3, and -9 cleavage were analyzed with western blotting. GAPDH was used as a loading control.

Given the significant role of mitochondria in apoptotic pathway [29], we investigated whether treatment with aumdubin induced mitochondrial apoptosis in lung cancer cells. As shown in Fig. 3A–C, we employed the cell-permeant dye Rhodamine123 (Rh123) to probe mitochondrial membrane potential (MMP) and found that aumdubin treatment led to the loss of MMP in a dose-dependent manner in A549 and H1299 cells. Moreover, we measured the dynamic changes of cytochrome c levels in cytoplasm and mitochondria by western blotting of isolated fractions, and found that aumdubin treatment increased the levels of cytochrome c in cytosol while deceased its levels in mitochondria (Fig. 3D, E). These findings indicated the release of cytochrome c from the mitochondria into the cytoplasm, which contributes to the formation of apoptosome, a multiprotein caspase-activating complex. In addition, Annexin-V FITC and propidium iodide (PI) double staining were applied on A549 and H1299 cells that were exposed to escalating concentrations of aumdubin, followed by detection with fluorescence microscopy. We found that aumdubin treatment increased apoptotic (i.e., Annexin-V/PI-positive) population in a dose-dependent manner and induced the morphological changes of apoptosis (Fig. 3F, G). We next analyzed the activation of caspases, a family of cysteine proteases that act as the main executioners of apoptosis. As shown in Fig. 3H–K, the active forms of caspase-3 and -9 as well as PARP cleavage were increased after aumdubin treatment in both dose- and time-dependent manners in A549 and H1299 cells. Collectively, these data indicate that aumdubin induces mitochondrial apoptosis in A549 and H1299 cells.

### DUB inhibition contributes to the anticancer activity of aumdubin

**Fig. 4:**
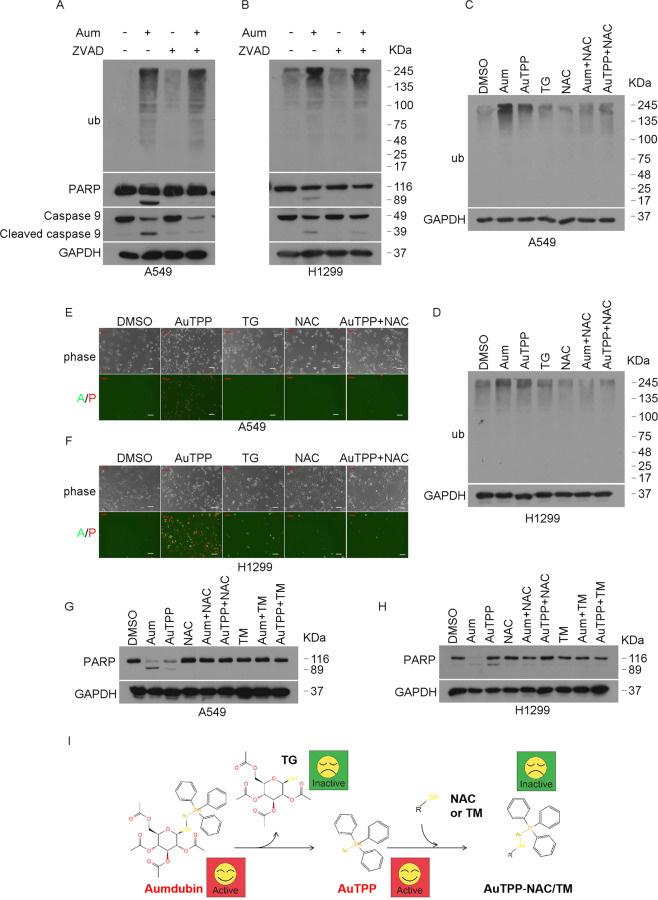
DUB inhibition contributes to the apoptosis-inducing effects of aumdubin in A549 and H1299 cells. **A**, **B** Z-VAD-FMK blocks aumdubin-induced apoptosis, but not accumulation of ubiquitinated protein. A549 (**A**) and H1299 (**B**) cells were incubated with 1 μM aumdubin (Aum) in the absence or presence of 50 μM Z-VAD-FMK (ZVAD) for 24 h, and then ubiquitinated proteins, PARP and caspase-9 cleavage were detected using western blotting. GAPDH was used as a loading control. **C**, **D** AuTPP but not TG induces the accumulation of ubiquitinated protein. A549 (**C**) and H1299 (**D**) were treated with the indicated doses of aumdubin, AuTPP, TG in the absence or presence of 5 mM NAC for 6 h, ubiquitinated proteins were detected using western blotting. **E**, **F** NAC inhibits AuTPP-induced apoptosis. A549 (**E**) and H1299 (**F**) were treated with aumdubin in the absence or presence of 5 mM NAC for 24 h, and then cells were stained with Annexin V-FITC/propidium iodide (A/P) and imaged under a fluorescent microscope. The typical fluorescent images were shown. Scale bars, 50 µm. **G**, **H** NAC and TM inhibits aumdubin and AuTPP-induced apoptosis. A549 (**G**) and H1299 (**H**) were treated with the indicated doses of aumdubin or AuTPP in the absence or presence of NAC or TM for 24 h, PARP was detected using western blotting. **I** Potential binding reaction between NAC or TM and aumdubin. Aumdubin may dissociate into TG and AuTPP. NAC or TM may bind with AuTPP to form new products, which lose the ability to induce apoptosis and DUB inhibition.

To understand the role of DUB inhibition in aumdubin-induced apoptosis, we first pre-treated A549 and H1299 cells with pan-caspase inhibitor z-VAD-FMK (zVAD) and tested its effects on aumdubin-induced caspase 9 activation, PARP cleavage, and ubiquitinated protein accumulation by western blotting. As shown in Fig. 4A, B, zVAD can completely reverse the cleavage of caspase 9 and PARP induced by aumdubin, but does not dramatically affect accumulation of ubiquitinated proteins, indicating aumdubin-mediated DUB inhibition happened prior to the induction of cell death or they might be two unrelated processes.

The chemical structure of aumdubin contains two parts, gold containing AuTPP and the thiol sugar moiety ligand (TG). To investigate the roles of AuTPP and TG, A549 and H1299 cells were treated with the indicated doses of aumdubin, AuTPP, or TG. It was clear that aumdubin had stronger activity in the induction of ubiquitinated protein accumulation than AuTPP while TG had no effect (Fig. 4C, D). Further, Annexin V/PI-staining experiment supports that AuTPP part of aumdubin rather than TG part played a crucial role in the inhibition of DUBs (Fig. 4E, F). Since most of the DUBs (e.g., USPs and UCHs) are thiol proteases [30], using thiol-containing agents might block the effects of active site inhibitors of thiol DUBs. We then tested the effects of NAC (*N*-acetyl-cysteine) and TM (tetrathiomolybdate), two thiol-containing agents, on DUB inhibition and cell death induced by aumdubin. We found that both NAC and TM reversed aumdubin/AuTPP-induced accumulation of ubiquitinated proteins (Fig. 4C, D), cell death (Fig. 4E, F), and PARP cleavage (Fig. 4G, H). These data suggest that aumdubin might be dissociated into inactive TG and active AuTPP inside the cells (Fig. 4I), and NAC or TM may bind with AuTPP to form a new product, which inhibits the effects of aumdubin on A549 and H1299 cells. Collectively, DUB inhibition contributes to the apoptosis-inducing activity of aumdubin.

### USP30 inhibition sensitizes cancer cells to aumdubin

**Fig. 5:**
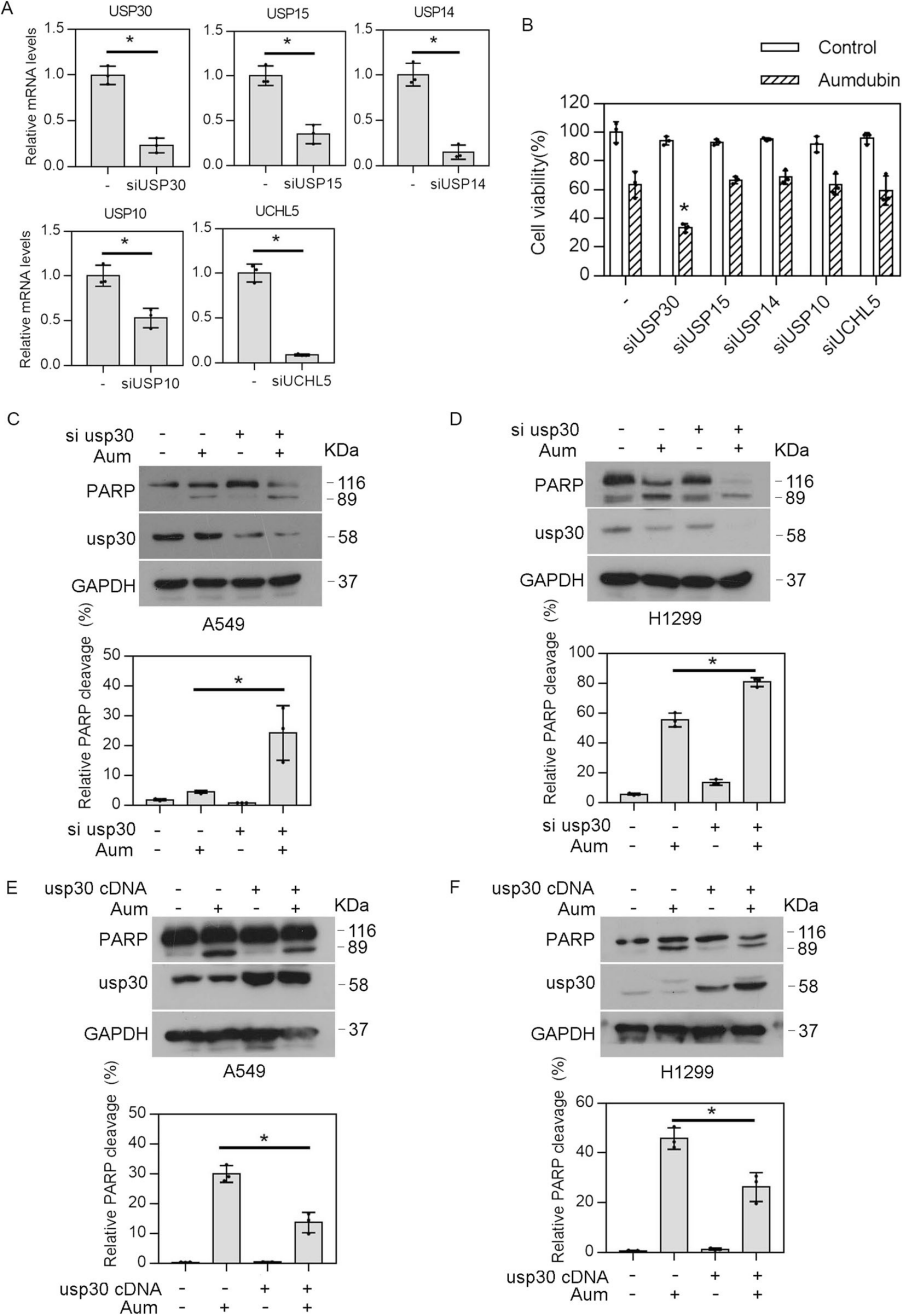
USP30 inhibition sensitizes cancer cells to aumdubin in lung cancer cells. **A** H1299 cells were transfected with Control siRNA or the indicated DUBs siRNA for 48 h, then mRNA expression of the indicated DUBs was measured by RT-qPCR and its expression level relative to the control was calculated. Mean ± SD (*n* = 3), ^*^P < 0.05, versus control. **B** Knockdown of USP30 enhances aumdubin-induced cell death. H1299 cells were transfected with Control siRNA or the indicated DUBs siRNA for 24 h, followed by treatment with or without 1 μM aumdubin (Aum) for 24 h. The cell viability was measured by using MTS assay. Mean ± SD (*n* = 3), ^*^P < 0.05, versus aumdubin alone. **C**, **D** Knockdown of USP30 enhances aumdubin-induced apoptosis. A549 (**C**) or H1299 (**D**) cells were transfected with Control siRNA or USP30 siRNA for 24 h, followed by treatment with or without 1 μM aumdubin (Aum) for 24 h. **E**, **F** Overexpression of USP30 attenuates aumdubin-induced apoptosis. A549 (**E**) or H1299 (**F**) cells were transfected with vector or usp30 cDNA plasmids for 24 h, followed by treatment with or without 1 μM aumdubin for 24 h. PARP and USP30 were analyzed with western blotting. The relative quantification of cleavage PARP/total PRAP were shown. GAPDH was used as a loading control.

We next asked whether aumdubin’s strong apoptosis-inducing activity is related to its potent inhibition on DUBs. We first knocked down one of the several DUBs, including USP30, USP15, USP14, USP10, and UCHL5 in H1299 cells (Fig. 5A). We found that only the knockdown of USP30, but not of any other DUBs, enhanced the aumdubin-mediated cytotoxicity (Fig. 5B). These findings suggest that inhibition of USP30 may contribute to aumdubin-induced apoptotic cell death. Indeed, knockdown of USP30 by using siRNA increased the levels of PARP cleavage in aumdubin-treated A549 and H1299 cells (Fig. 5C, D). In contrast, overexpression of USP30 alleviated PARP cleavage in aumdubin-treated A549 and H1299 cells (Fig. 5E, F). These results indicate that inhibition of USP30 sensitizes cancer cells to aumdubin-induced apoptosis in lung cancer cells.

### Aumdubin induces apoptosis by regulating ubiquitination of Bax protein

**Fig. 6:**
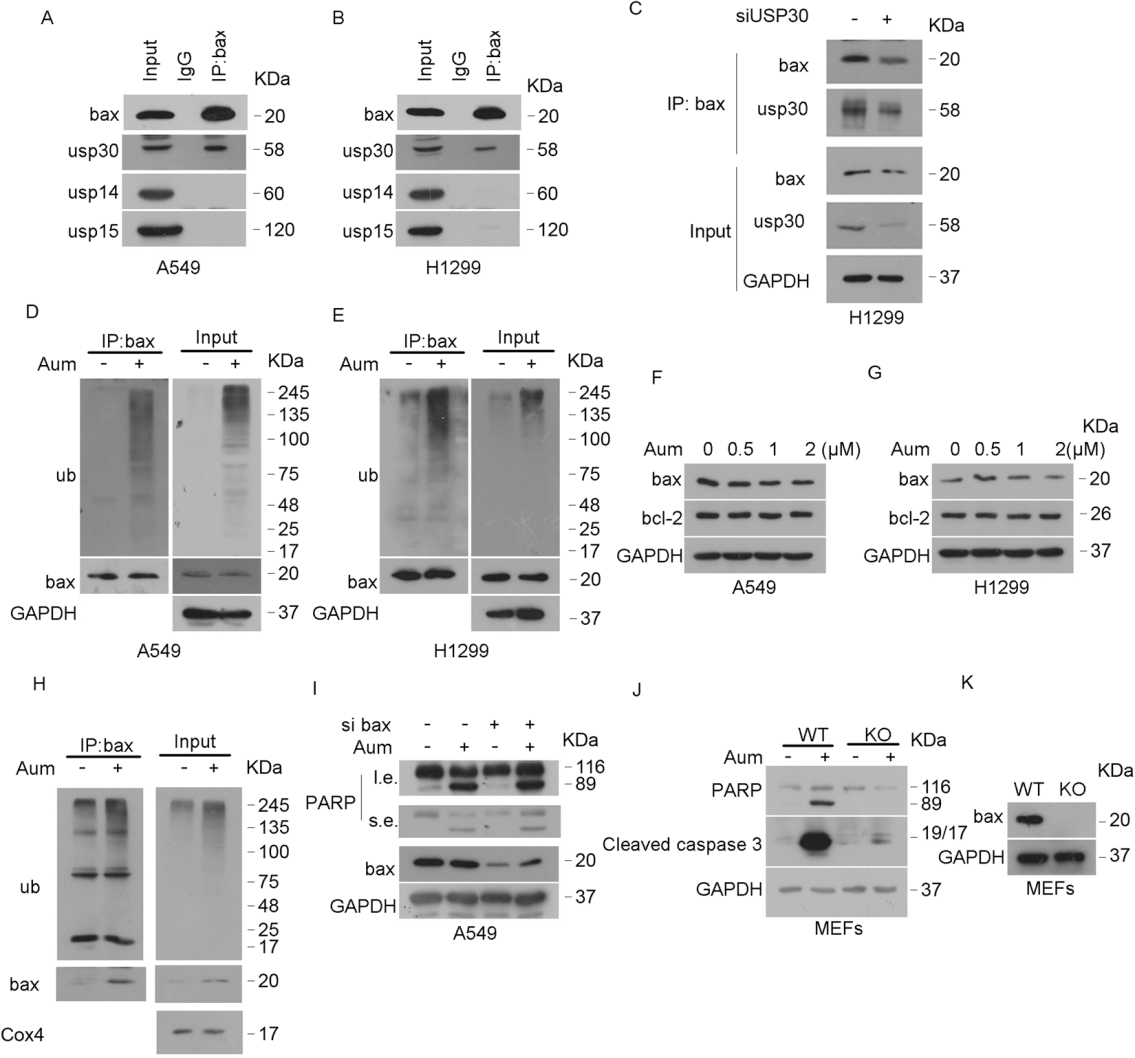
Aumdubin induces apoptosis by regulating ubiquitination of Bax. **A**, **B** USP30 interacts with Bax. Endogenous Bax were immunoprecipitated from A549 (**A**) and H1299 (**B**) cells using an anti-Bax antibody, co-immunoprecipitated proteins were detected using western blotting. **C** USP30 specifically binds Bax. Endogenous Bax were immunoprecipitated from USP30 knockdown or control and H1299 cells using an anti-Bax antibody, co-immunoprecipitated proteins were detected using western blotting. **D**, **E** Aumdubin induces ubiquitination of Bax. A549 (**D**) and H1299 (**E**) cells were treated with1 μM aumdubin (Aum) for 6 h, and then endogenous Bax were immunoprecipitated, followed by immunoblotting using anti-ubiquitin antibody. **F**, **G** Aumdubin does not induce degradation of Bax and bcl-2. A549 (**F**) and H1299 (**G**) cells were treated with various concentrations of aumdubin for 12 h, Bax and bcl-2 were detected with western blotting assay. **H** Aumdubin increases the mitochondrial Bax and ubiquitination of mitochondrial Bax. H1299 cells treated with or without 1 μM aumdubin for 6 h, then endogenous Bax were immunoprecipitated from the mitochondrial fractions, followed by immunoblotting using anti-ubiquitin antibody. **I** Knockdown of Bax alleviates aumdubin-induced apoptosis. A549 cells were transfected with Control siRNA or Bax siRNA for 24 h, followed by treatment with or without 1 μM aumdubin for 24 h, PARP and Bax were detected by western blotting. **J**, **K** Knockout of Bax blocks alleviates aumdubin-induced apoptosis in MEFs. Wild-type and Bax/Bak double-knockout MEFs were treated with or without 1 μM aumdubin for 24 h. PARP and caspase-3 cleavage were detected by western blotting.

It is well known that Bax plays a key role in apoptosis by inducing the loss of mitochondrial membrane’s outer membrane permeability, which contributes to the subsequent cytochrome c leaking into the cytoplasm [31]. We wonder whether USP30 may regulate the (de)ubiquitination of Bax protein. We analyzed the physical interaction of USP30 with Bax by co-immunoprecipitation (co-IP). As shown in Fig. 6A, B, endogenous USP30, but not other DUBs such as proteasomal USP14 and mitochondrial USP15, was detected in the Bax affinity-isolation precipitates of A549 and H1299 cells, indicating that USP30 can specifically bind with Bax. As expected, knockdown of USP30 decreased the binding between USP30 and Bax protein in H1299 cells (Fig. 6C). We next examined the ubiquitination status of Bax during aumdubin-induced cell death. We found that the ubiquitination of Bax was increased after aumdubin treatment in A549 and H1299 cells (Fig. 6D, E). As ubiquitination contributes to the degradation of proteins, we then analyzed the expression of Bax, as well as antiapoptotic protein Bcl-2 in aumdubin-treated cells, and found that aumdubin did not alter the expression of Bax and Bcl-2 in A549 and H1299 cells (Fig. 6F, G). Consequently, the increased ubiquitination of Bax induced by aumdubin did not contribute to the degradation of Bax protein. Instead, we found that aumdubin increased the levels of both mitochondrial Bax and ubiquitinated mitochondrial Bax in H1299 cells (Fig. 6H), indicating that ubiquitination of Bax may enhance its mitochondrial location, which is responsible for its apoptosis induction ability.

We then investigated whether Bax participates in aumdubin-induced apoptosis. Knockdown of Bax expression using siRNA reduced PARP cleavage induced by aumdubin in A549 cells (Fig. 6I). To further clarify the role of Bax in aumdubin-induced apoptosis in cancer cells, wild-type and Bax/Bak-deficient MEF cells were treated with aumdubin for 24 h. We found that Bax/Bak-deficient MEFs were resistant to aumdubin treatment compared to wild-type MEF cells (Fig. 6J, K). Taken together, we conclude that aumdubin induces apoptosis in a Bax-dependent manner.

## Discussion

Since the reports of systematic investigations of the anticancer potential of gold compounds started appearing in the 1980s [32], a wide range of gold compounds with promising anticancer properties have been investigated over the past several decades, which were categorized in terms of phosphane gold (I) compounds, gold thiolates, gold porphyrins, gold compounds with bipyridyl-type ligands, organogold species—gold N-heterocyclic carbenes, cyclometallated gold complexes, and gold alkenyls [33]. The general modes of action exhibited by gold compounds include direct DNA damage, dysfunction of mitochondria, cell cycle arrest, topoisomerase inhibition, 20S proteasome inhibition, specific kinase inhibition, and inhibition of other transcriptional factors [34]. Auranofin, for example, a gold (I)-containing phosphine compound, which is considered to cause TrxR inhibition, both the cytosolic form TrxR1 and the mitochondrial form TrxR2 [35], as well as proteasome DUB inhibition [24, 36], displays promising anticancer properties in a range of carcinomas. In this study, we explored the anticancer property of a novel gold compound, aumdubin, which is chemically modified by substituting triethylphosphine of auranofin with TPP. Importantly, aumdubin shows stronger anticancer activity than auranofin against lung cancer cells both in vitro and in vivo. However, TrxR may not be the main target of aumdubin, since aumdubin fails to induce ROS and aumdubin-mediated cell death cannot be reversed by ROS scavenger vitamin C.

In addition, we investigated whether aumdubin displayed mitochondria-targeting capacity due to TPP’s strong mitochondria-targeting ability. Mitochondria are important intracellular organelles, which play key roles in cellular proliferation and death. Some notable differences between mitochondria of cancer cells and normal cells have been identified. These include that cancer cells have significantly higher MMP and levels of ROS, compared with normal cells [37]. Indeed, after treatment with aumdubin, mitochondrial transmembrane potential was destroyed in A549 and H1299 cells. Furthermore, we found that aumdubin was able to inhibit USP30, a mitochondrial outer membrane DUB. These findings were consistent with the hypothesis that aumdubin could target mitochondria, where it inhibits the activity of USP30. One potential shortcoming of aumdubin is that it also inhibits other DUBs, including USP14, USP10, and UCHL5. How to develop specific DUB inhibitors, especially that used in vivo, remains a major challenge. For example, Maria et al. tested the specificity of nine previously reported DUB inhibitors (e.g., USP1 inhibitor pimozide and USP7 inhibitor P22077) against a group of 32 DUBs [38]. Unfortunately, none of these compounds showed selectivity towards a single DUB, but unselectively inhibited most DUBs [38]. However, although aumdubin can inhibit several DUBs, aumdubin-induced cell death may be related to USP30 inhibition, but not inhibition of other DUBs (Fig. 5B). Since CRISPR drop-out screening has not indicated USP30 for being important for cell survival [39], several other mechanisms may be involved in aumdubin-induced apoptosis. It is also difficult to determine whether aumdubin-mediated tumor suppression in vivo is due to its DUB inhibition activity. Although DUB activity can be measured from tissue by using the Ub-derived active-site-directed probes [40], it is not clear if lysis procedure would displace the DUB inhibitor from its targets. Although we found that aumdubin induced accumulation of ubiquitinated proteins and speculated that this is caused by DUB inhibition, we still lack direct evidence of aumdubin in DUB inhibition in vivo.

Apoptosis induction is a promising strategy for anticancer therapy. There are many anticancer drugs targeting various stages of apoptosis, one of which is stimulation of proapoptotic molecules [4]. In this work, we found that aumdubin induced Bax-dependent apoptosis. Moreover, we found interaction between Bax and USP30, as well as aumdubin-increased ubiquitination and mitochondrial location of Bax. Several E3 ligases (e.g., parkin and IBRDC2) have been reported to regulate the ubiquitination of Bax [8, 9], however, the DUB that is required for deubiquitination of Bax remains unclear. Since UPS30 mainly localizes on the outer mitochondrial membrane, in the future, it will be of interest to determine whether the ubiquitinated proteins in aumdubin-treated cells accumulate on the outer mitochondrial membrane. A plausible explanation is that USP30 is the DUB of Bax, and USP30 inhibition-mediated Bax ubiquitination induces the translocation of pro-apoptotic Bax on the mitochondria. However, the precise mechanisms remain to be elucidated. We found that aumdubin induced the increase of total ubiquitinated protein (Fig. 1D, E). Future studies should investigate whether USP30 plays a role in regulating ubiquitination of non-mitochondrial proteins.

In conclusion, as a novel auranofin derivative, aumdubin exhibits strong mitochondrial USP30-inhibiting ability and substantial anticancer capacity.

## Materials and methods

### Chemicals and antibodies

Aumdubin was synthesized in our laboratory. Other agents used include auranofin (Enzo Life Sciences International Inc.; BML-EI206-0100); bortezomib (S1013), b-AP-15 (S4920) and pan-caspase inhibitor Z-VAD-FMK (S7023) (Selleck Chemicals); *N*-acetyl-*L*-cysteine (A7250), tetrathiomolybdate (323446), Rh123 (R8004), Chloro (triphenylphosphine) gold(I) (AuTPP, 254037) (Sigma-Aldrich Inc.); 1-Thio-beta-D-glucose tetraacetate (TG, Alfa Aesar Inc.; L11404.03). Antibodies used in this study were purchased from following sources: antibody against ubiquitin (P4D1) (Santa Cruz Biotechnology; sc-8017); usp1 (8033), usp10 (8501), usp14 (11931), usp15 (66310), bcl-2 (15071), Bax (5023), cyto c (4280), cleaved caspase 9 (20750), caspase 3 (9662), cleaved caspase 3 (9661), PARP (9532), cox4 (4850), and β-actin (4970) (Cell Signaling Technology); Bax used for MEFs (60267-1-Ig), usp2 (10392-1-AP), usp28 (17707-1-AP) (Proteintech Group); uchl5 (Abcam; ab176377); usp30 (Sigma-Aldrich Inc.; SAB4503385); and GAPDH (Bioworld Technology; AP0063).

### Cell lines and cell culture

Human lung cancer cell lines A549 and H1299 were purchased from American Type Culture Collection (ATCC), wild-type and Bax/Bak double-knockout MEFs were obtained from Dr. Quan Chen (the Group of Apoptosis and Cancer Biology, the State Key Laboratory of Biomembrane and Membrane Biotechnology (GACB), Institute of Zoology, Chinese Academy of Sciences). Lung cancer cell lines (A549, H1299) and MFEs were grown in RPMI 1640 and DMEM, respectively, supplemented with 10% FBS. Cultured cells were maintained at 37 °C and 5% CO_2_.

### Measurement of reactive oxygen species generation

ROS production was detected as previously reported [23]. Cancer cells were treated with aumdubin (1 μM) in the absence or presence of antioxidant vitamin C for 1 h. The cells were harvested and incubated with the free serum medium with addition of DCFH-DA (10 μM) for 20 min at 37 °C in the dark. In the presence of ROS, DCFH penetrates the cells and is in turn oxidized to DCF. DCF fluorescence was detected by flow cytometry.

### Co-immunoprecipitation (co-IP) assay

A549 or H1299 cells were lysed in cell lysis buffer (Cell Signaling Technology, 9803) and quantified with BCA (Thermo Fisher Scientific Inc., 23225), Dynabeads m-270 Epoxy (Invitrogen, 14311D) coupled with indicated antibodies were prepared based on the manufacture’s instruction. They were mixed equally and rotated at 4 °C for 2 h. After washing by wash buffer, the antibodies–lysate mixtures were suspended with 3x loading buffer and boiled at 70 °C for 10 min. The EP tube was placed on a magnetic mount and supernatant was collected for immunoblotting.

### Isolation of mitochondria and western blotting

Mitochondrial protein extraction kit (KeyGEN BioTECH, KGP850) was used for mitochondrial fractionation isolation following the manufacture’s instruction. Western blotting was carried out as previously described [41]. In brief, an equal amount of protein extracts was fractionated by 12% SDS-PAGE and electrically transferred onto a polyvinylidene difluoride (PVDF) membrane (Millipore). Primary antibodies and appropriate horseradish peroxidase-conjugated secondary antibodies were used to detect the designated proteins. The bound secondary antibodies on the PVDF membrane were reacted to the ECL detection reagents (Santa Cruz Biotechnology) and detected by exposing to X-ray films (Kodak, Japan).

### DUB active-site-directed labeling assays

This was performed as previously described [42]. Briefly, after treatment with aumdubin, whole-cell lysates were prepared in DUB buffer (25 mM Tris-HCl pH 7.4, 5 mM MgCl_2_, 20 mM NaCl, 200 μM ATP). Following quantification and then incubation with HA-ub-vs for 1 h at 37 °C, they were boiled in the reducing sample buffer and fractionated via SDS-PAGE. After transfer to PVDF membranes, HA-ub-vs-labeled DUBs were immunodetected by western blotting.

### USP30 activity assays

The activity of USP30 was determined by the increase in fluorescence measured as a result of the enzyme catalyzed cleavage of the fluorogenic substrate Ubiquitin-AMC (BostonBiochem, Cambridge, MA, USA) generating Ubiquitin and AMC. USP30 enzyme assay was performed by preincubating 20 nM of USP30 with the indicated concentration of aumdubin in the assay buffer (50 mM Tris-HCl [pH 7.5], 250 mM sucrose, 5 mM MgCl2, and 1 mM PMSF) for 15 min and then incubated with Ub-AMC substrate in a 100 μL reaction volume for 1 h at 25 °C. AMC released from substrate cleavage was recorded with a microplate reader (Varioskan Flash 3001, Thermo).

### Cell viability assay

MTS assay (CellTiter 96Aqueous One Solution reagent; Promega, Shanghai, China) was used to test cell viability. In all, 1 × 10^5^/mL cells in 100 μL were treated with the indicated concentrations of compounds for 24 h, control cells received DMSO for a final concentration the same as the highest concentration of compounds but less than 0.1% v/v; 3 h before culture end, 20 μL MTS was added to the wells. The absorbance density was read on a 96-well plate reader at wavelength 490 nm.

### Cell death assay

Apoptosis was determined by Annexin V-fluoroisothio-cyanate (FITC)/PI double staining assay (KeyGEN BioTECH, KGA108) according to previous description [43]. Cells were grown to 70% confluence in 24-well plates, followed by treatment with aumdubin or other indicated compounds for 24 h. Then cell death was assessed by staining with Annexin/PI double staining in dark for 30 min at 37 °C in situ and recorded under an inverted fluorescence microscope.

### Mitochondrial membrane integrity measurement

The MMP of aumdubin- or DMSO-treated cells was analyzed using Rhodamine-123 staining. Cells were treated with various concentrations of aumdubin for 12 h and stained with 2.5 μM of rhodamine-123 for 30 min at 37 °C in the dark. Following the staining, the cells were washed with 4 °C PBS and then recorded under an inverted fluorescence microscope.

### Transfection assay

Control siRNA (sc-37007), USP30 siRNA (sc-96007), and Bax siRNA (sc-29212) were purchased from Santa Cruz Biotechnology, USP30 cDNA (NM_032663.4) was purchased from GeneCopoeia. Cells were plated in dishes for 24 h, grown to 60–70% confluence. All siRNAs were used at a final concentration of 25 nM and usp30 cDNA at 1 ng, transfected into cells with Lipofectamine™ 3000 Transfection Reagent according to manufacturer’s instructions (Invitrogen, L3000015). After 24 h, the medium was replaced with fresh RPMI 1640 containing 10% fetal bovine serum for further analysis.

### Nude mouse xenograft model

All animal protocols used were approved by the Institutional Animal Care and Use Committee of Guangzhou Medical University. The BALB/c-nude mice were obtained from Guangdong Laboratory Animal Monitoring Institute and housed in barrier facilities with a 12 h light–dark cycle, with food and water available ad libitum. To generate murine subcutaneous tumors, 250 × 10^4^ of A549 cells in 100 µL phosphate-buffered saline (PBS) were inoculated subcutaneously on the right flank of 5-week-old male nude mice. At 72 h after inoculation, mice were randomly allocated to either control or treatment group. The mice were treated with control (DMSO, cremophor, and normal saline at 1:3:6 ratio, v-v-v), auranofin (8.8 μmol/kg/day, intraperitoneal injection), or aumdubin (8.8 μmol/kg/day, intraperitoneal injection) for 13 days, respectively. Tumors were measured every other day with calipers. Tumor xenografts were removed afterwards, weighed, fixed, and stored.

### Immunohistochemistry and hematoxylin-eosin staining

Formalin-fixed xenografts and organs (heart, liver, spleen, lung, and kidney) of mice were embedded in paraffin and sectioned at 4 µm. Tumor xenograft sections were immunostained using the MaxVision kit (Maixin Biol) according to the manufacturer’s instructions. The primary antibodies were used as indicated. To each slide, 3.5 μL MaxVisionTM reagent was applied. Color was developed with 0.05% diaminobenzidine and 0.03% H_2_O_2_ in 50 mM Tris-HCl (pH 7.6), and the slides were counterstained with hematoxylin. A negative control for every antibody was also included for each xenograft specimen by substituting the primary antibody with preimmune serum. The organs (heart, liver, spleen, lung, and kidney) were stained with hematoxylin and eosin for histopathologic evaluation.

### Statistical analysis

All the results were expressed as mean ± s.d. where applicable. GraphPad Prism 7.0 software (GraphPad Software, San Diego, CA, USA) was used for statistical analysis. Differences between two groups were evaluated for statistical significance using two-tailed Student’s *t*-test. For testing differences among three or more independent groups, one-way analysis of variance, followed by the Holm-Sidak test for pair-wise comparisons, were performed. *P*-value of <0.05 was considered statistically significant. No statistical methods were used to predetermine sample sizes, but our sample sizes are similar to those generally employed in the field.
